# Effects of orientation and structure on solar radiation interception in Chinese solar greenhouse

**DOI:** 10.1371/journal.pone.0242002

**Published:** 2020-11-06

**Authors:** Demin Xu, Yiming Li, Yue Zhang, Hui Xu, Tianlai Li, Xingan Liu

**Affiliations:** 1 College of Horticulture, Shenyang Agricultural University, Shenyang, China; 2 National & Local Joint Engineering Research Center of Northern Horticultural Facilities Design & Application Technology (Liaoning), Shenyang, China; 3 College of Engineering, Shenyang Agricultural University, Shenyang, China; Soil and Water Resources Institute, GREECE

## Abstract

In order to further improve the utilization of solar energy in Chinese Solar Greenhouse (CSG), this paper systematically studied the effects of orientation and structure on solar radiation interception in CSG. A solar radiation model has been developed based on the previous research, which taking solar motion law, meteorological data, and optical properties of materials into consideration. The established model was used to optimize the orientation and structure of CSG. The analysis of structure considered two major structural parameters, which are the ridge height and the horizontal projection of the rear roof. Moreover, the widely used Liao-Shen type Chinese solar greenhouse (CSG-LS) has been taken as the prototype in the present research, and the measured data of the typical clear day was used for the model validation. The results showed that the ridge height has a remarkable influence on the solar energy captured by CSG-LS. Compared with the optimization of a single factor, the comprehensive optimization of orientation and structure can increase the solar radiation interception of the rear wall by 3.95%. Considering the limiting factor of heat storage-release capacity and the shading effect on the greenhouse structure, the optimal lighting construction of the CSG-LS (with a span of 9.0 m) was specified as 7~9° from south to west of azimuth angle, 4.5~4.7 m ridge height, and 1.4~1.6 m horizontal projection of the rear roof at 42°N latitude. The proposed solar radiation model can provide scientific guidance for the CSG-LS construction in different areas.

## Introduction

In recent years, crop production in Chinese solar greenhouse (CSG) has become the most profitable agricultural industry, which has made a historic contribution to the supply of vegetables and fruits in the cold regions of northern China [1, 2]. As the most important energy source in CSG production, sunlight is not only the fundamental prerequisite for the plant photosynthesis [3], but also one of the necessary conditions to accomplish CSG overwintering production. With the consumption of the Earth's natural resources and the deterioration of the climate environment, solar energy is expected to be the most used energy source by 2050 [4]. As a renewable energy source, the use of solar energy plays a crucial role in keeping sustainability, which, in turn, is the key to meeting the developmental needs of future generations [5–8]. However, as a typical efficient utilization industry of solar energy, solar energy utilization in CSG production is far from sufficient [9]. The heating of solar greenhouse mostly depends on fossil fuels which can lead it to play a significant role in environmental pollution, energy consumption, and operational cost [10–12]. Compared with the use of heating devices in the solar greenhouse, the optimized greenhouse can save a lot of natural resources and reduce the operating cost of the greenhouse during the winter season. There are many factors affecting the interception of solar radiation in CSG, such as orientation, structure, and distribution rule of plant population, etc. Among them, the orientation and structure of CSG are decisive factors for controlling the amount of solar energy captured.

To explore the effect of greenhouse orientation on the spatial distribution of light, a computer model has been developed for calculating the direct solar light transmission into a single-span greenhouse, but the model neglected the reflected light of plastic film and the effect of polarization [13, 14]. Considering the optical properties of plastic film, as the orientation of CSG in Shenyang is 5~6° from south to west, the sunlight inflow from lighting roof reaches the maximum, which is about 0.3% higher than that of facing the due south, but the solar radiation intercepted by the ground and wall inside the greenhouse is difficult to be calculated [15]. The solar radiation that enters the greenhouse through the lighting roof not only provides a suitable light environment for crops to grow, but also is stored by the wall and ground in the form of heat. When there is no supply of solar radiation at nighttime, the heat stored during the daytime is released to keep the greenhouse environment stable and meet the basic temperature requirements for crop survival. In other words, the ability of the wall and ground to intercept solar radiation should be regarded as an indicator in greenhouse construction. On this basis, the different orientation of greenhouses has been studied using a mathematical model based on solar radiation transmission, and it was found that the E-W orientation is preferred for even-span or modified arch shape greenhouses [16]. According to the calculation results of a computational model, the orientation of CSG should be south by west in northern China [17]. However, these models lack consideration of the effect of structure on the greenhouse light environment. Taking the greenhouse shape parameters and materials’ optical properties into consideration, a solar radiation allocation and spatial distribution model of CSG was established to optimize the light environment. Furthermore, it was found that the ridge height and the horizontal projection of the rear roof are the major structural parameters in the structure of CSG affecting the solar energy capture. But this model could not optimize the orientation [18]. After synthetically analyzing the influence of different orientations and structures on capturing solar energy and saving heating cost [19], it can be found that the E-W orientation of a greenhouse and elliptically curved surface aspect ratio equal to 4 was most suitable for a northern tropical region. The orientation and structure also can be determined by three-dimensional shadow analysis. What needs to be noticed is that the amount of solar radiation cannot be quantified [20].

In current literature, most of the researches in the optimization of the greenhouse light environment ignore the influence of the optical physical parameters. Moreover, there are great differences between Chinese solar greenhouses and foreign domed greenhouses. The structure of the CSG is more complex, which is composed of the lighting roof, wall, roof, and sidewalls [18]. All of these are factors that affect the achievement of maximum solar radiation interception and enhancement of the nighttime temperature in CSG. However, most of the current studies on structural optimization of CSG are focused on a single influencing factor [21]. Due to the lack of comprehensive analysis of the influential factors and the meteorological conditions, the reasonable size of each part of CSG can not be determined, thus the structural optimization of the whole greenhouse can not be realized. Most of the CSG structures and orientations are only determined by the limited field experience without going through robust design procedures. Thus, it is beneficial to use novel approaches to improve the design of CSG.

In this investigation, a novel solar radiation model has been developed numerically to analyze the influence of azimuth angle, ridge height, and horizontal projection of rear roof on the solar energy interception of CSG. These three influential factors are the decisive parameters of the shape and orientation for CSG. In the actual CSG construction process, due to the geographical location and site constraints, it is impossible for farmers to build the greenhouses in a consistent size. Therefore, the optimal conclusion of this study on the greenhouse size is to determine a reasonable range rather than a set of optimal size data. In comparison to previous models [22, 23], this model has taken the optical physical parameters of the CSG and the meteorological data into consideration, which could improve the accuracy of the calculation. The quantitative statistics of solar radiation interception in greenhouse has been combined with resource conservation. The results can provide scientific and quantitative guidance for the CSG construction. Furthermore, the obtained results have great significance for the transformation of the old CSG.

## Materials and methods

### Experimental site and design

**Fig 1:**
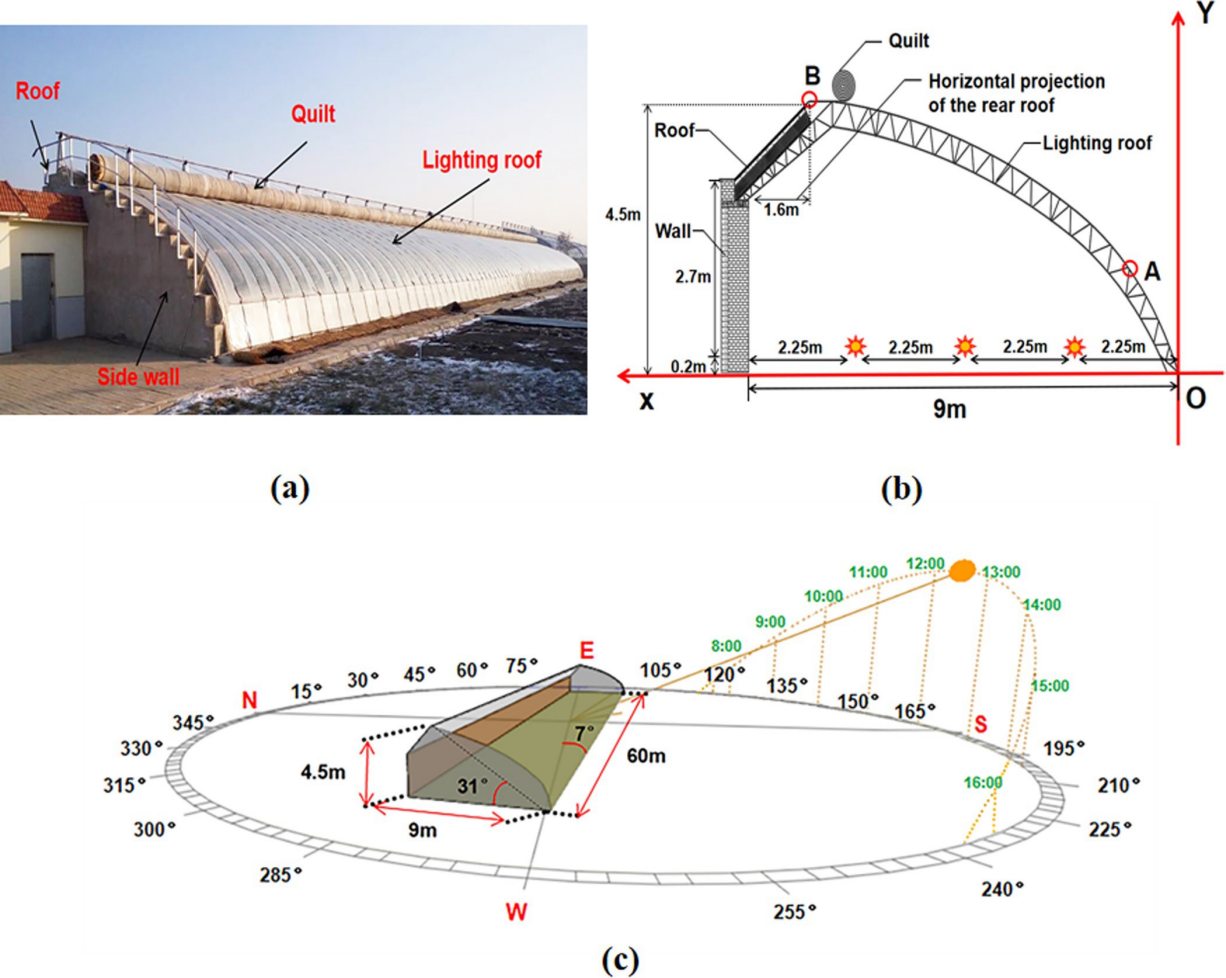
Schematic diagram of CSG-LS. (a) photographic view; (b) profile of experimental solar greenhouse; (c) three-dimensional view.

This study takes the widely used Liao-Shen type Chinese Solar Greenhouse (CSG-LS) as the prototype. The greenhouse is located in Shenyang Agriculture University, Liaoning Province, China (41.8°N, 123.4°E). Liaoning province is the birthplace of Liao-Shen type Chinese solar greenhouse. The structural parameters of CSG-LS are shown in Table 1. As presented in Fig 1, the azimuth of the greenhouse is 7° from south to west, and the dimensions of the greenhouse are a ridge height of 4.5 m, 2.7 m of the wall, 1.6 m of the horizontal projection of the rear roof, span 9.0 m, and length 60.0 m. It also contains an arched steel truss structure frame, covered with a 0.15 mm thick plastic film. On winter nights, the lighting roof is covered with the heat preservation quilt. To determine the optimal orientation and structure of the greenhouse, the three-dimensional greenhouse models with different structures and orientations were built based on controlling the span of the CSG-LS (9.0 m) to be consistent (Fig 1C). According to the previous experiments and investigations, the CSG-LS with a span of 9.0 m is more prominent in terms of internal temperature and solar radiation. The change range of orientation and structure of CSG-LS built by farmers according to their accumulated field experience is 10° south by east to 10° south by west, 4.3 m to 4.9 m ridge height, and 1.4 m to 2.0 m horizontal projection of the rear roof. To further determine a reasonable range and facilitate the model analysis, the orientation and structure were divided into different parts by equidistant. Then the influences of azimuth angle, ridge height, and horizontal projection of rear roof on the solar energy interception of these greenhouses were quantitatively analyzed by using the established solar radiation model. The evaluation index is the amount of solar energy captured on each surface of the greenhouse at different time periods on the winter solstice. In the northern hemisphere on the winter solstice, the solar altitude angle is the smallest, and the solar radiation intensity is relatively weak, so it is representative to choose the winter solstice as the analysis period.

**Table 1:** Chinese solar greenhouse building materials reference index.

Material	Transmittance	Reflectance	Specific heat capacity	Heat conductivity
	(%)	(%)	(J·kg^-1^·K^-1^)	(W·m^-1^·K^-1^)
Wall (brick)	-	6	1051	0.50
Roof (wood)	-	20	2510	0.29
Soil	-	12	1010	0.85
Plastic film	70	15	1600	0.19

### Theoretical foundation

#### Establishment of the lighting roof curve

Previous researches have shown that the cumulative difference of solar radiation is insignificant for CSG lighting roof with similar shapes [24]. The present study also ignores the slight radian difference between a mathematical curve and a real greenhouse skeleton, and employs the mathematical model of the lighting roof curves of CSG. Two key points are selected as presented in Fig 1B. One is at the front embankment corner of CSG, which is at a critical height for manual operation, and the position of point A is fixed at (0.7 m, 1.5 m). The other is the highest point of the greenhouse, where the position of point B is changeable. The set-up equations are as follows:

$$Y_{1}=aX_{1}^{b}$$ (1)

$$Y_{2}=aX_{2}^{b}$$ (2)

$$b=(\text{ln}Y_{2}-\text{ln}Y_{1})/(\text{ln}X_{2}-X_{1})$$ (3)

$$a=Y_{2}/X_{2}^{b}$$ (4)

where (*X*_1_, *Y*_1_) and (*X*_2_, *Y*_2_) refer to the coordinates of point A and point B, respectively. According to the coordinates of point A and point B, the values of the coefficient a and b can be easily obtained by solving the simultaneous equations. By substituting the values of the coefficient a and b into the equation of *Y* = *aX*^*b*^, the greenhouse lighting roof curve can be got [25].

#### Solar energy captured on the lighting roof

According to the above lighting roof curve equations, the formula for calculating the total solar energy captured on the lighting roof surface (*I*_*T*_) is given by:

$$I_{T}=\int_{0}^{x_{2}}\sqrt{1+{\left(abx^{b}{}^{-1}\right)}^{2}}dx\times L\times I_{L}{}_{R}$$ (5)

where *x*_2_ refers to the horizontal distance from the point B to the front embankment corner of CSG (m); the value of *a* and *b* can be obtained from Eqs 1–4. L represents the length of CSG (m); *I*_*LR*_ stands for the average amount of solar radiation intercepted by the lighting roof of CSG (W/m^2^). According to the difference of environmental meteorological conditions, the solar radiation intercepted by the lighting roof of CSG (*I*_*LR*_) can be divided into two cases, one is the solar radiation intercepted by the lighting roof in the sunny day (*I*_*LRS*_), the other is the solar radiation intercepted by the lighting roof in the cloudy day (*I*_*LRC*_). The value of *I*_*LRS*_ and *I*_*LRC*_ can be calculated using Eqs 6 and 7.

$$I_{LRS}=I_{n}[\sin h\cos\theta +\cos h\sin\theta \cos(A_{1}-\alpha )]+I_{s}(1+\cos\theta )/2$$ (6)

$$I_{LRC}=I_{LRS}\times CCF$$ (7)

In the above equations, *I*_*LRS*_ consists of two parts (Eq 6), one is solar direct radiation and the other is solar diffuse radiation [26]. Where *I*_*n*_ is the amount of solar direct radiation on the normal plane (W/m^2^); *I*_*s*_ stands for the amount of solar diffuse radiation on the normal plane (W/m^2^); *h* and *A*_1_ respectively refer to the solar altitude angle and solar azimuth angle (°); *θ* and *α* represent the lighting roof angle and azimuth angle of CSG, respectively (°). The value of *θ* and *α* can be measured in the field. When the solar greenhouse is facing south, the value of *α* is equal to 0°. For the solar greenhouse facing east, the value of *α* is negative. On the contrary, if the solar greenhouse facing west, the value of *α* is positive. Furthermore, the value of *h*, *A*_1_, and *CCF* can be calculated as follows [27]:

σ=arcsin{0.39785sin(4.896+0.0172J+0.03345sin(6.224+0.0172J)]} (8)

sinh=sinφsinσ+cosφcosσcos[15(t-12)] (9)

$$\sin A_{1}=\cos\sigma \sin[15(t-12)]/\cos h$$ (10)

$$CCF=P+Q(CC)+R{\left(CC\right)}^{2}$$ (11)

where σ is the degree of the solar declination angle (°); *J* stands for the day of the year, when the value of *J* is known, σ can be easily obtained by using Eq 8. *φ* refers to the geographic latitude of the greenhouse (°); and *t* represents the decimal hours on the 24-hour clock. By substituting the value of σ, *φ*, and *t* into Eq 9, the value of *h* can be calculated. Taking a similar approach, the value of *A*_1_ also can be calculated by Eq 10. *CCF* refers to the cloud cover coefficient; *P*, *Q*, and *R* all are constants, and the value of these constants is related to the seasons. In winter, the value of *P*, *Q*, and *R* are 1.14, 0.003, -0.0082, respectively. *CC* stands for the number of clouds [28]. The solar direct radiation on the normal plane can be calculated by setting up the equations as follows [29]:

$$I_{n}=(I_{0}/r^{2})P_{1}{}^{csch}$$ (12)

$$r=r_{n}/r_{0}{}^{-1}$$ (13)

where *I*_0_ refers to the solar radiation constant, the value of *I*_0_ is 1353 W/m^2^; *r* stands for the variable radius of the Earth, and the value of *r* can be calculated by Eq 13, *r*_*n*_ represents the actual distance of the Earth from the sun on a given day; *r*_0_ is the average distance of the Earth from the sun throughout the year. *P*_1_ represents the atmospheric transparency coefficient. By substituting Eqs 9 and 12 and the value of *P*_1_ into Eq 14, the value of the solar diffuse radiation on the normal plane (*I*_*S*_) can be calculated as follow [30]:

$$I_{S}=1.2\times I_{n}\times \sin h\times (1-P_{1})\times (1-P_{1}{}^{csch})/(1-1.4\times \text{ln}P_{1})$$ (14)

#### Solar radiation intensity in CSG

The solar radiation entering the solar greenhouse is mainly divided into two parts, one is the solar energy captured by the wall (*I*_*W*_), the other is the solar energy captured by the ground (*I*_*g*_). The transmissivity of the lighting roof is one of the factors that influence the amount of solar energy captured in CSG. The solar energy captured by the wall and ground can be calculated by Eqs 15–18, respectively [18].

$$I_{w}=[I_{n}\cos h\cos(A_{1}-\alpha )]\times \tau_{D}+(I_{S}/2)\times \tau_{S}$$ (15)

$$I_{g}=I_{n}\times \sin h\times \tau_{D}+I_{S}\times \tau_{S}$$ (16)

$$\tau_{D}=\tau_{\beta }\times (1-r_{1})\times (1-r_{2})\times (1-r_{3})$$ (17)

$$\tau_{S}=\tau_{d}\times (1-r_{1})\times (1-r_{2})\times (1-r_{3})$$ (18)

Where *τ*_*D*_ is the direct light transmittance; *τ*_*S*_ stands for the diffuse light transmittance. By substituting Eqs 10–14 and the value of *τ*_*D*,_ *τ*_*S*_ into Eq 15, the value of the solar energy captured by the wall (*I*_*W*_) can be obtained. According to the obtained results from Eqs 9, 12 and 14, the value of the solar energy captured by the ground (*I*_*g*_) can be calculated. The value of *τ*_*D*_ and *τ*_*S*_ can be given by Eqs 17 and 18. Among them, *τ*_*β*_ stands for the transmittance at the incident angle of *β*. *τ*_*D*_ is expressed as clean pervious to light material of scattered light transmittance. *r*_1_, *r*_2_, and *r*_3_ refer to the light transmission loss caused by the opaque materials, aging of plastic film, dust and water droplets, respectively.

#### Time for the heat preservation quilt to be opened and closed

The opening and closing time of the heat preservation quilt is one of the key factors to determine whether the solar greenhouse can achieve multi-season vegetable production. They directly affect the stability of heat balance inside the solar greenhouse and the illumination duration of crops. When the geographic latitude of the greenhouse (*φ*) is known, the opening time (*T*_*O*_) and closing time (*T*_*C*_) of quilt can be easily calculated by Eqs 19 and 20 [17].

$$T_{O}=7.3\varphi -215.4$$ (19)

$$T_{C}=2.8\varphi -76.1$$ (20)

## Results

### Validation of the present model

**Fig 2:**
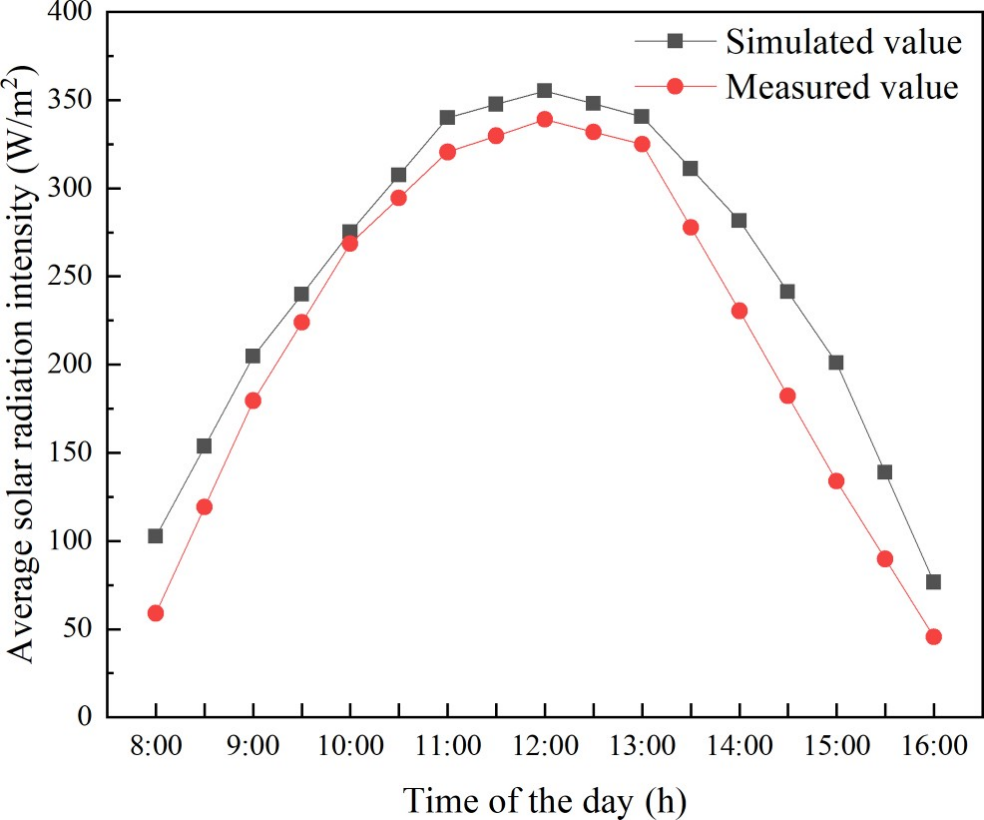
Comparison between simulated value and measured value.

In order to verify the established model, a sunny day near the winter solstice (December 23, 2019) was selected to measure the near-ground solar energy interception in CSG-LS. The data were measured by MP-200 handheld Pyranometer, with a measurement range between 0 and 1999 W/m^2^, accuracy of ± 5%, and response time < 1ms. The validation of the present model has used three measurement points so that the accuracy of the collected data can be improved. The layout of measurement points is shown in Fig 1B. The distance between them is 2.25 m. The measurement points are 0.2 m above the greenhouse ground. This greenhouse is an empty greenhouse with no crops, and the heat preservation quilt is not covered at night. According to the calculation result by Eqs 19 and 20, the opening and closing time of heat preservation quilt in Shenyang area are close to 8:00 a.m. and 16:00 p.m. respectively. Therefore, the time period for simulating and measuring solar radiation data is selected from 8:00 a.m. to 16:00 p.m. On the observation day, the data were collected every 0.5 hours and measured two times. The directional order of data collection is from north to south and then measured in reverse. In this way, the time difference can be eliminated, and the average value was taken to represent the value of that point. The predicted values of solar energy interception near the ground in CSG-LS are calculated using Eq 16. The measured values of solar energy interception near the ground show that the intensity of solar radiation decreases gradually from south to north. Since the difference between the measured data is not significant, the average value of the measured data at these three measurement points is used to represent the solar radiation intercepted by the ground. As presented in Fig 2, the predicted values are consistent with the overall variation trend of the measured values. The determination coefficient (*R*^2^) is used to check the accuracy of the model, of which the value is 0.96768, indicating that the data of the solar radiation model of CSG is highly reliable. The simulated values are slightly higher than the experimental measurement. This is because the aging degree of plastic film at different positions of the lighting roof is not consistent. But in the calculation process, the aging degree of plastic film is taken as an average value, which is underestimated compared with the actual aging degree.

### Influence of azimuth on the light environment of CSG

**Fig 3:**
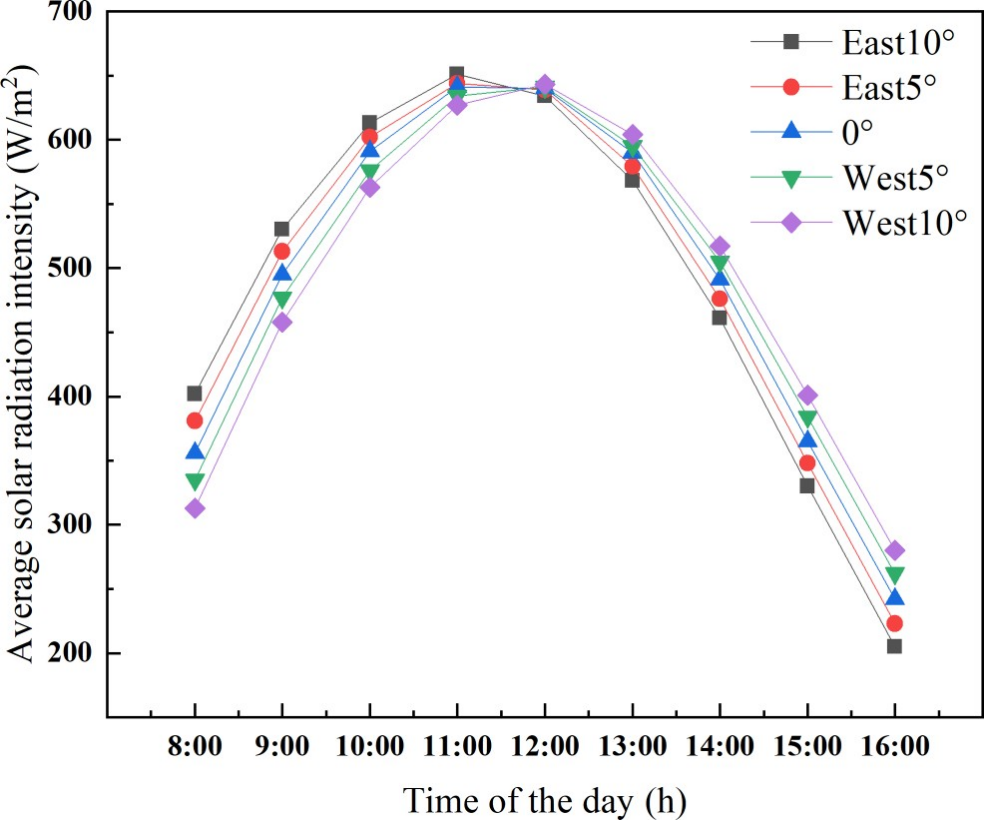
Average daily solar radiation intensity of CSG-LS at different azimuth angles.

Winter is not only the season in which CSG receives the shortest illumination, but also the season in which heat is most needed. In order to determine the optimal azimuth angle of CSG in Shenyang, as presented in Fig 3 and Table 2, five azimuth angles (i.e. 10° from south to east, 5° from south to east, 0°, 5° from south to west and 10° from south to west) were calculated on the winter solstice. The different azimuth angles are respectively abbreviated in the figure (i.e. East 10°, East 5°, 0°, West 5°, West 10°). The peak of captured solar energy in CSG facing by west appears at 12:00 p.m., which occurs one hour later than those facing east. When all the solar energy interception of the lighting roof reach the peak value, the maximum difference between the CSG with different orientations is 11 W/m^2^. Moreover, the solar energy interception capacity of CSG facing west is significantly higher than that of CSG facing east during the time period from 1:00 p.m. to 4:00 p.m. According to the principle that the solar radiation intensity is strongest at 12:00 p.m., CSG should be oriented in such a way as to maximize the interception of solar energy at noon. Therefore, the obtained results show that the appropriate range of the azimuth angle is 5~10° from south to west.

**Table 2:** The maximum solar radiation intercepted in Chinese solar greenhouse with different orientations.

Time of the day	maximum value (W/m^2^)				
	East10°	East5°	0°	West5°	West10°
11:00	651	644	640	634	627
12:00	634	639	639	641	643

**Fig 4:**
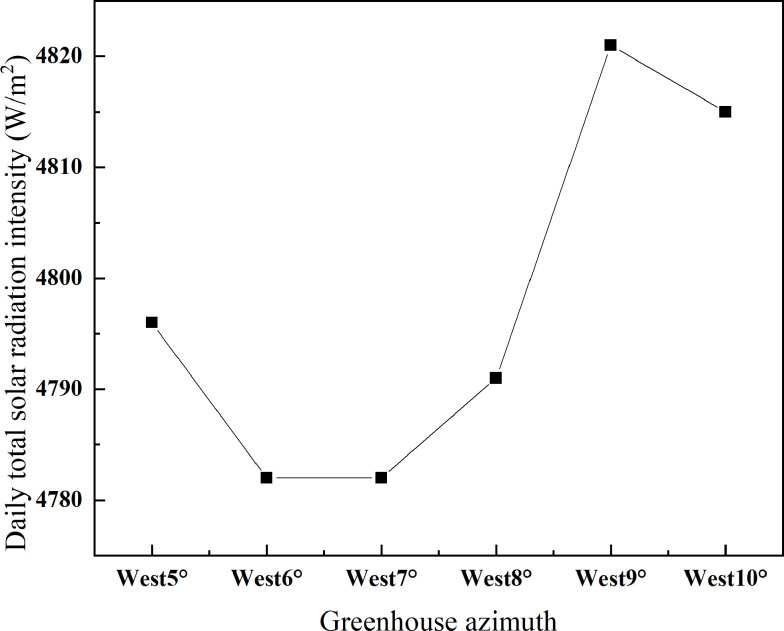
Cumulative solar radiation intensity of CSG-LS at different azimuth angles.

Then, in order to further determine the optimal orientation of the greenhouse, the cumulative solar energy captured by CSG-LS at different azimuths is used as an evaluation index. As presented in Fig 4, the cumulative captured solar energy reaches the maximum when the azimuth is 9° from south to west. Taking the outdoor temperature in winter into consideration, to ensure the stability of environmental change in the greenhouse, the heat preservation quilt should not be lifted until the captured solar energy is enough to compensate the heat lost from the lighting roof. According to the calculation result by Eq 19, the time of lifting the heat preservation quilt in Shenyang usually should not be earlier than 8:30 a.m. during the winter. Considering the aspect of geophysics, the lighting roof of CSG facing due south in Shenyang can receive the radiation of direct sunlight at 8:00 a.m. The azimuth of the greenhouse changes by 1° to the west and the time for the lighting roof to receive solar radiation is delayed by 4 minutes [17]. Therefore, the azimuth of greenhouse in Shenyang should be greater than 7° from south to west, so that it can extend the illumination time. In conclusion, the reasonable azimuth angle of CSG in Shenyang (*φ* = 42°) is 7~9° from south to west.

### Influence of ridge height on the light environment of CSG

**Fig 5:**
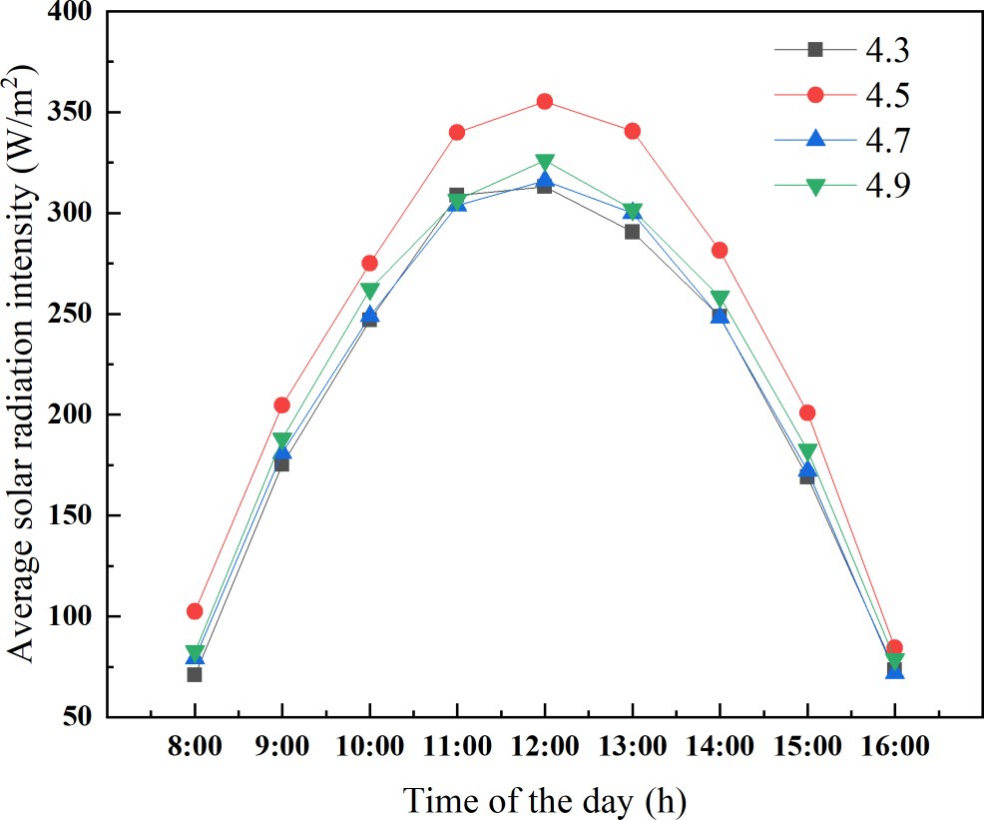
Solar radiation intensity of the ground in CSG-LS with different ridge heights.

As presented in Fig 5, in order to determine the reasonable ridge height range of CSG-LS, the greenhouse models with different ridge heights of 4.3 m, 4.5 m, 4.7 m, and 4.9 m were respectively established for simulation analysis. The winter solstice was chosen as the simulation object. The solar energy interception of CSG-LS ground with a ridge height of 4.5 m is significantly higher than that of the other greenhouses. The maximum difference in solar radiation intensity between them reaches 49.92 W/m^2^. However, there is no significant difference in the solar energy interception of the greenhouse with ridge heights of 4.3 m, 4.7 m, and 4.9 m.

**Fig 6:**
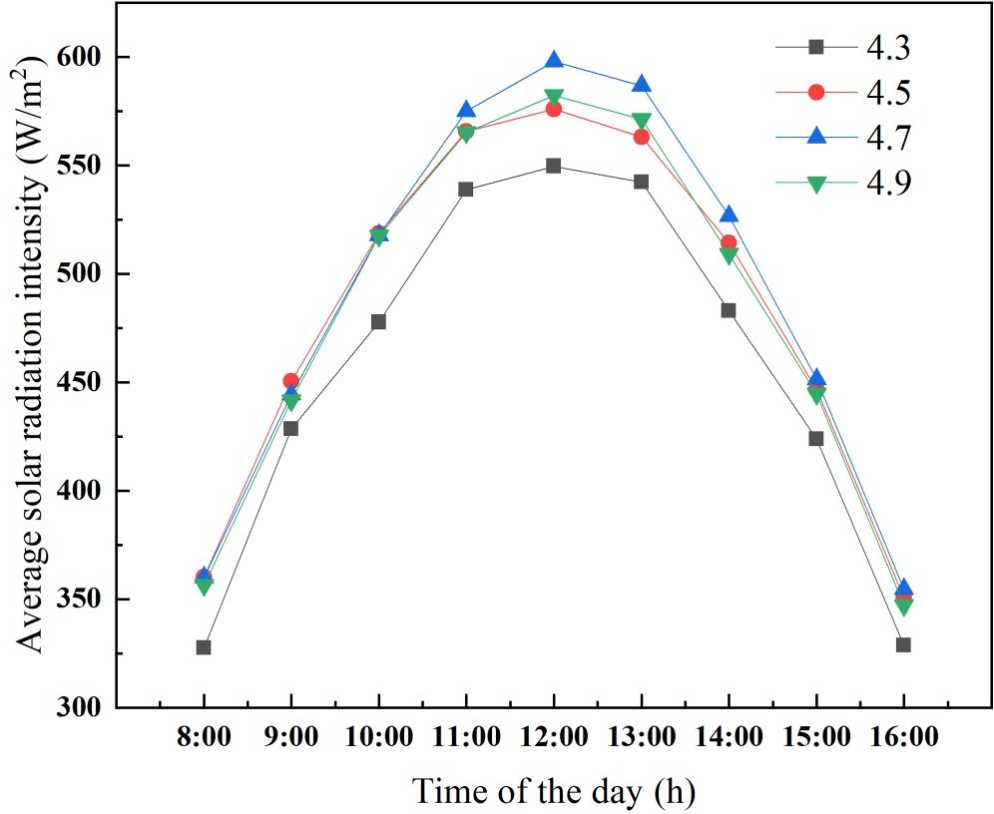
Solar radiation intensity of the wall in CSG-LS with different ridge heights.

The change of ridge height of CSG-LS also makes a difference in the solar energy interception of the wall. As presented in Fig 6, there is an insignificant difference in solar energy interception between the walls of greenhouses with different ridge height before 11:00 a.m. From 11:00 a.m. to 2:00 p.m., the solar energy interception of the wall of 4.7 m ridge height is significantly higher than the others, and the maximum solar radiation intensity is 48.27 W/m^2^, which is 8.78% higher than that of the greenhouse with 4.3 m ridge height.

**Fig 7:**
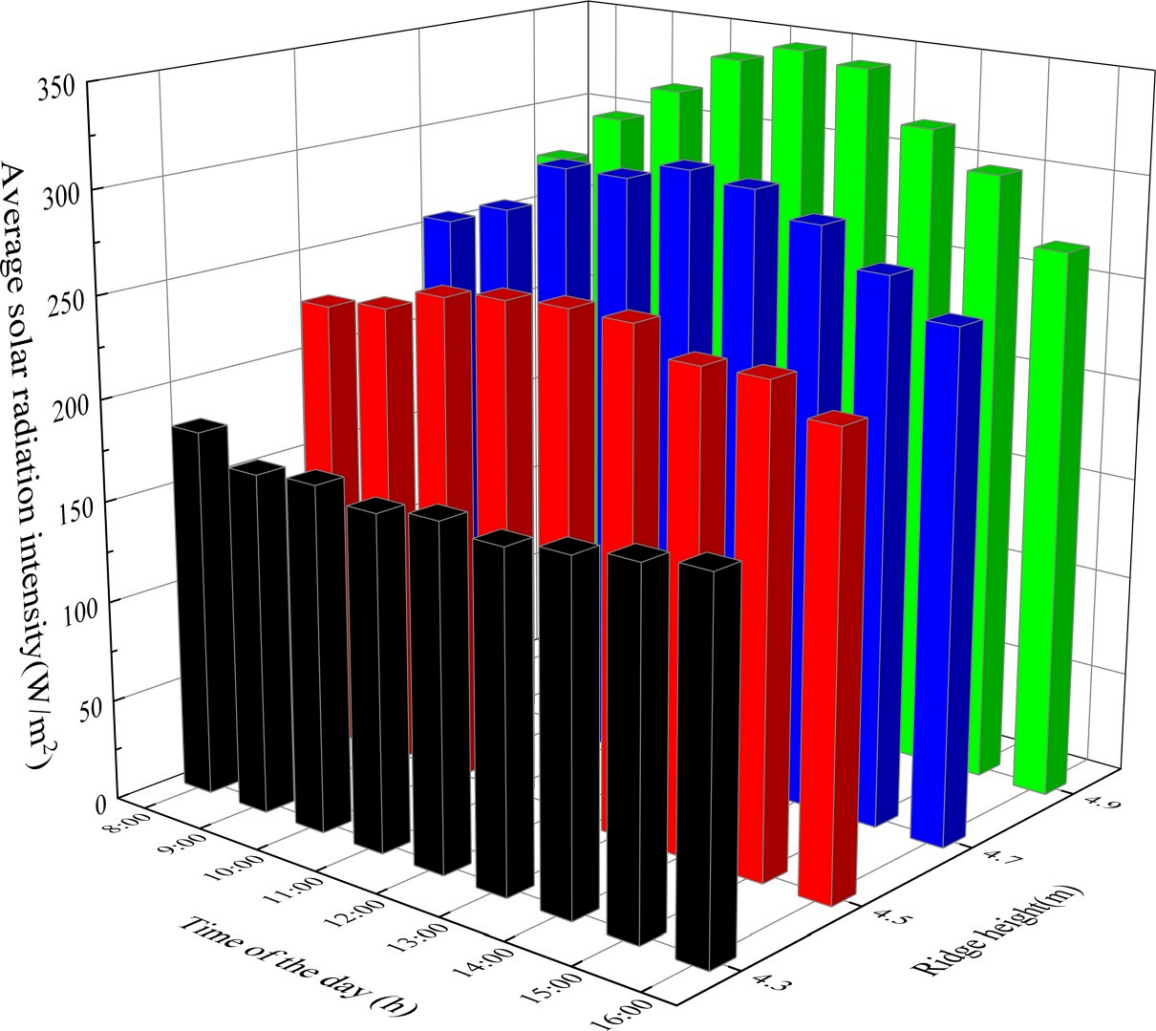
Solar radiation intensity of the roof in CSG-LS with different ridge heights.

The solar energy interception of the roof of CSG-LS changes most obviously with the increase of ridge height. As presented in Fig 7, the solar energy interception of the roof is linearly correlated with the ridge height. The maximum solar energy interception difference on the roof of the four greenhouses reaches 178.51 W/m^2^. However, the inner layer of the roof in CSG-LS is usually made of wooden boards with high specific heat capacity. It only has a limited influence on insulation performance, while its heat storage performance is poor [31]. Therefore, when the influence of changing ridge height or changing horizontal projection of the rear roof on solar energy interception inside the greenhouse is analyzed, the solar energy intercepted by the roof can be ignored.

**Fig 8:**
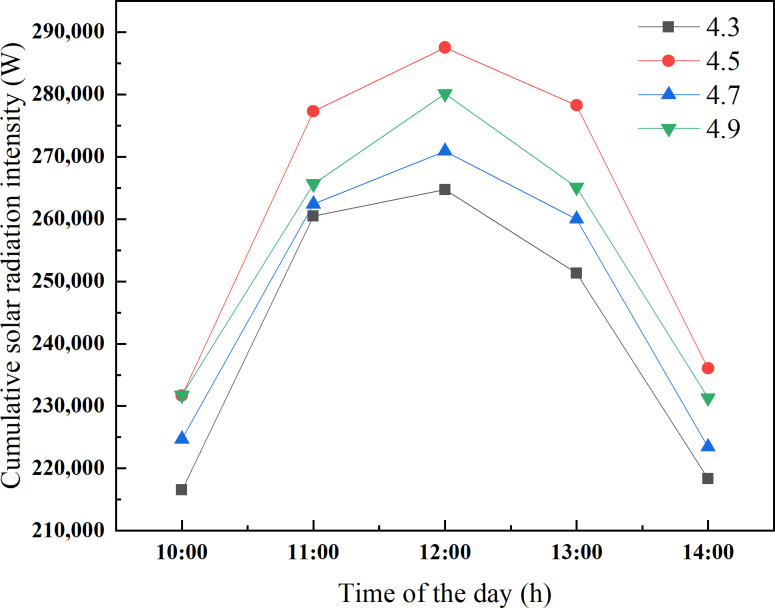
Cumulative solar radiation intensity of different ridge height of CSG-LS.

To further determine an optimal range of ridge height, the amount of solar energy captured by the ground and the wall are added together from 10 a.m. to 2 p.m., which can be used as a measure index of greenhouse lighting and heat storage effect. This period is the maximum heat storage period of the CSG. As presented in Fig 8, the total solar radiation intercepted by the CSG with a ridge height of 4.5 m is significantly better than that of the others. Taking the intercept of the total solar radiation into consideration, the ridge height of CSG-LS should be maintained at about 4.5 m. In consideration of actual production, the main heat of the greenhouse at night comes from the wall due to the shielding effect of crops on the ground of CSG-LS. If the ridge height of CSG is too high, the thermal insulation ratio becomes small and the thermal insulation property becomes worse. Therefore, according to Figs 6 and 8, it can be clear that the upper limit of ridge height should be 4.7m instead of 4.9m. In summary, the ridge height of CSG-LS should be maintained at 4.5~4.7 m.

### Influence of horizontal projection of rear roof on the light environment of CSG

**Fig 9:**
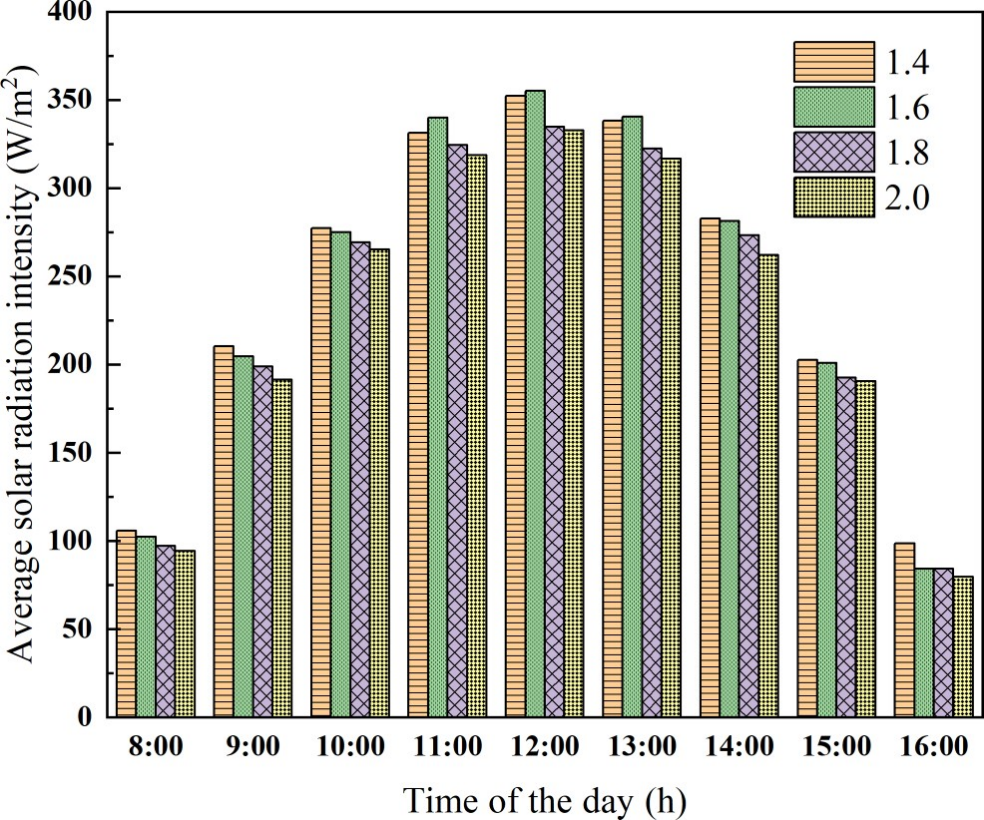
Solar radiation intensity of the ground with different horizontal projection of rear roof.

In addition to the ridge height, another important factor affecting the light environment of the greenhouse is the horizontal projection of rear roof. However, the horizontal projection of the rear roof has a relatively weak influence on the light environment in the greenhouse compared with the ridge height. Therefore, on the basis of the ridge height of 4.5 m, the CSG models with the horizontal projections of the rear roof (i.e. 1.4 m, 1.6 m, 1.8 m, and 2.0 m) were respectively established to analyze the indoor light environment. The time period of numerical simulation was also set on the winter solstice. As presented in Fig 9, the influence of the greenhouses with different horizontal projection on the solar radiation interception of ground shows that 1.4~1.6 m is higher than 1.6~2.0 m, and the maximum solar radiation intensity difference between greenhouses reaches 23.86 W/m^2^.

**Fig 10:**
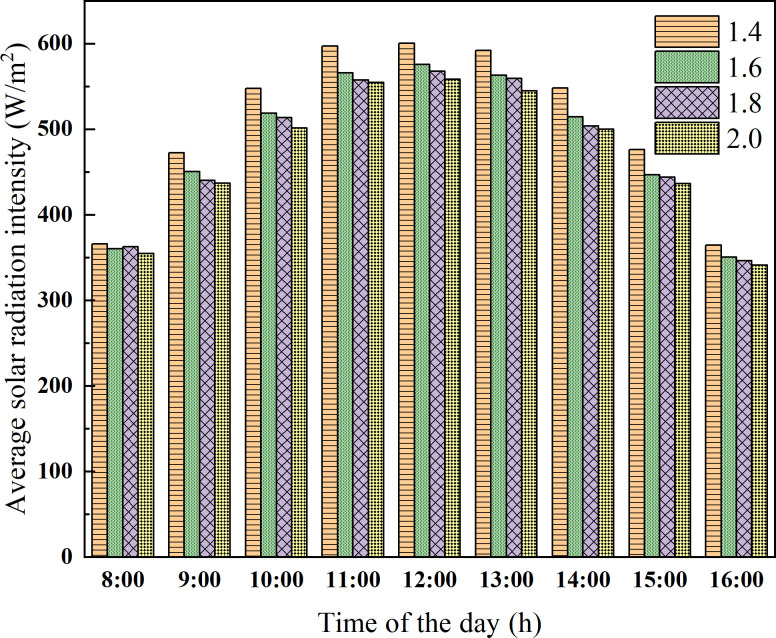
Solar radiation intensity of the wall with different horizontal projection of rear roof.

As presented in Fig 10, on the winter solstice, the solar radiation interception of the wall with a horizontal projection of 1.4 m is the highest. The maximum solar radiation intensity difference reaches 47.35 W/m^2^. The solar radiation intercepted by the walls of the greenhouse with a horizontal projection of 1.6 m and 1.8 m only has a little difference. But with the change of the solar movement track in different seasons, the long roof can affect the light transmittance of the greenhouse. Meanwhile, the long roof can increase the cost of building greenhouses. In conclusion, the reasonable range of the horizontal projection of the rear roof is 1.4~1.6 m.

### Optimizing evaluation and resource saving

**Fig 11:**
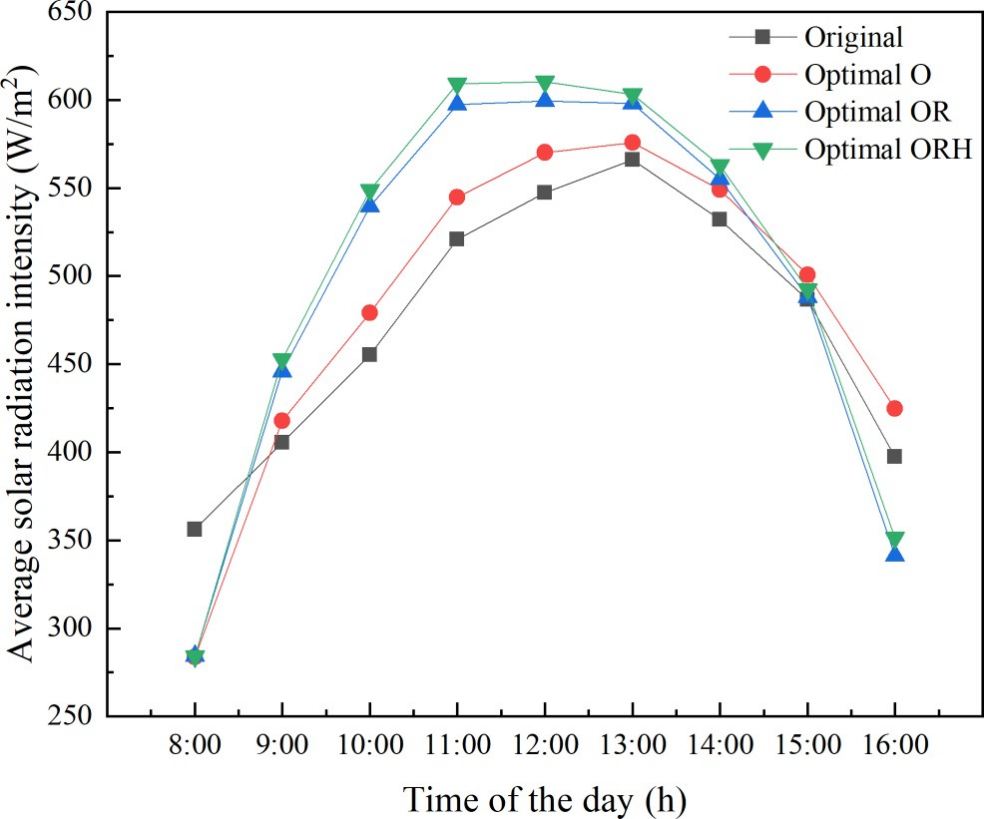
Solar radiation intensity of the wall with different greenhouses.

The construction of solar greenhouse should consider three important parameters, namely daylighting, heat storage, and heat preservation. Among them, there is a positive correlation between daylighting and heat storage. As mentioned above, the wall of CSG-LS is the most important part of daylighting and heat storage. Therefore, in order to quantitatively describe the priority of the comprehensive optimization factors, the solar radiation interception of the wall is used as the evaluation index for comparative analysis. According to the previous calculation results, a greenhouse with poor daylighting is selected for stepwise optimization, in which the orientation and structure are 6° from south to west, 4.3 m of ridge height, 2.0 m of the horizontal projection of the rear roof (Original). Subsequently, the orientation of the greenhouse is optimized. The azimuth changes from 6° south by west to 9° south by west (Optimal O). On this basis, the ridge height of the greenhouse is optimized and the height increases from 4.3 m to 4.7 m (Optimal OR). Finally, the horizontal projection of the rear roof is optimized, and the length reduces from 2.0 m to 1.4 m (Optimal ORH). As presented in Fig 11, compared with the optimization of a single factor, the comprehensive optimization of the factors that affect the daylighting of CSG-LS has a more significant effect on the enhancement of daylighting.

**Fig 12:**
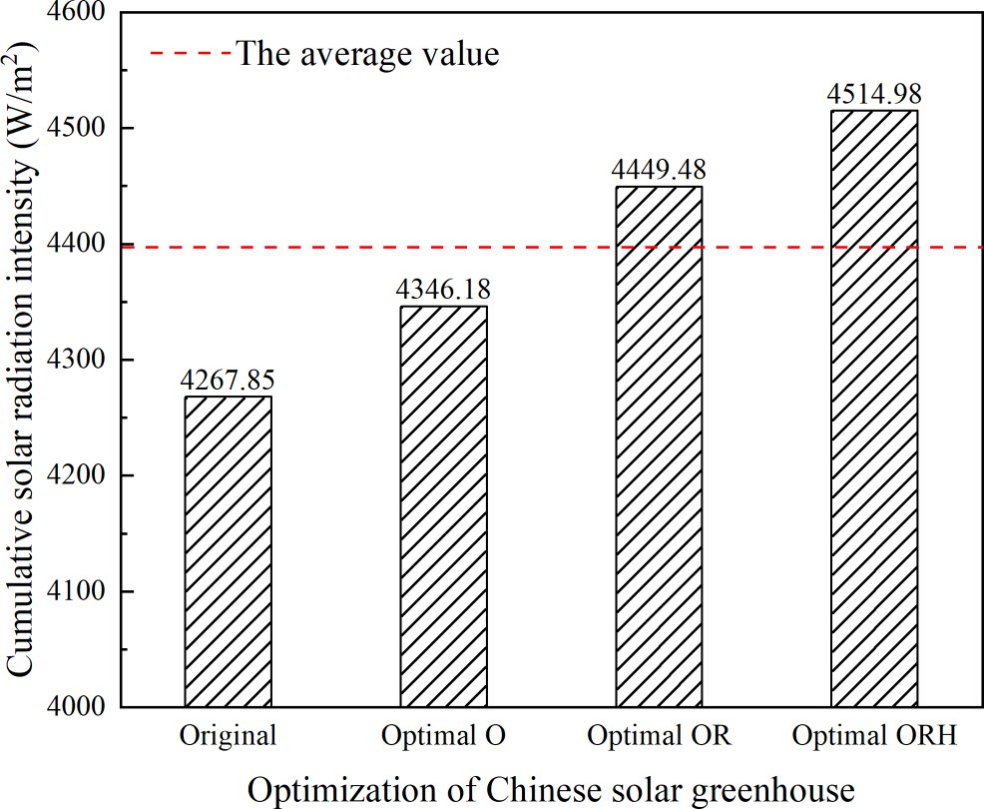
Solar radiation intensity of the wall with different greenhouses.

Furthermore, the daily cumulative solar radiation intensity of the wall is used to more clearly indicate the importance of a comprehensive analysis of the influencing factors. As presented in Fig 12, by optimizing the orientation of the greenhouse, the lighting effect of the wall can be improved by 1.84%. At the same time, the orientation and ridge height of the greenhouse are optimized, the lighting effect of the wall can be improved by 4.26%. When all three factors are optimized, the lighting effect of the wall can be improved by 5.79%. From this point of view, it is necessary to comprehensively optimize the factors that affect the daylighting of CSG.

In China, coal-fired hot air stoves are widely used to heat the solar greenhouses, its rated heat output is 11,630 W, the coal consumption is 8 kg per hour, and the operating power is 800 W. Now an approximate calculation analysis is made on the effect of energy-saving by the greenhouse in different azimuth angles. The difference between the intercepted solar energy by these greenhouses is significant. Based on the obtained results in Fig 4, it has been calculated that the maximum daily accumulative solar energy interception difference of the greenhouses at different azimuth angles is 29,160 W. By the empirical formula conversion [32], this is equivalent to the energy released by running this stove for 2.5 hours, and it can save about 20 kg of coal and 7,200 kJ of electricity. The heating time in the cold season is estimated at 60 days. The results indicate that adjusting the azimuth of the greenhouse could save 1,200 kg of coal and 432,000 kJ of electricity per year.

## Discussion

As can be seen from the obtained results, among the three factors studied in the present research, the ridge height of CSG-LS has a more significant impact on the interception of solar energy. The effect of the horizontal projection of the rear roof on the interception of solar energy is relatively weak. When the land resources are sufficient, the space between greenhouses can be appropriately extended. In this way, the ridge height can be appropriately increased on the basis of the reasonable horizontal projection of the rear roof, so as to achieve better daylighting and heat storage performance without shading. Meanwhile, the appropriate azimuth angle is of vital importance to ensure the duration of sunshine obtained by CSG-LS. It is very effective to optimize the greenhouse through this model, just by optimizing the orientation of the greenhouse can reduce the amount of coal used on cultivated land by 2.2 kg/m^2^ per year. According to statistics from the Department of Agriculture, China is the Country with the highest greenhouse-based vegetable production in the world. In 2010, the area of solar greenhouses has reached over 47 million ha [33]. If the captured solar energy is enough for heating solar greenhouse, there is no need to use a high running cost auxiliary heating system. Furthermore, an optimized greenhouse can not only mitigate energy consumption and environmental pollution but also maintain a stable environment for crops inside the greenhouse. In the present study, the solar radiation model established is based on the empty greenhouse, so that the interference of crops on experimental measurements can be eliminated. The influence of the height of wall and the length of span on solar energy interception can also be explored through the method proposed in this study. With the consumption of natural resources and the improvement of environmental awareness, the use of the orientation and structure optimization of solar greenhouse will become an inevitable trend.

## Conclusions

In this study, a relatively complete analysis model of the light environment of CSG was proposed to systematically analyze the effects of orientation and structure on solar radiation interception, which taking the optical physical parameters of greenhouse building materials, meteorological conditions, and other factors into consideration. Moreover, the established model has been verified successfully by the strong consistency between the simulated and measured values. Using the established model, the solar radiation intensity of each surface of CSG-LS at a given time has been calculated to determine the optimal orientation and structural parameters. According to the results of this study, the optimized ridge height has a more significant effect on the interception of solar energy. For Shenyang (*φ* = 42°), the optimal lighting construction of the CSG-LS (with a span of 9.0 m) is specified as 7~9° from south to west of azimuth angle, 4.5~4.7 m ridge height and 1.4~1.6 m horizontal projection of the rear roof. The optimization of solar energy interception capability in CSG-LS also can effectively reduce the consumption of non-renewable energy and protect the ecological environment.

## Supporting information

S1 Table Nomenclature. (DOC)
