# Supplementary material for: Effects of orientation and structure on solar radiation interception in Chinese solar greenhouse
Source: PLoS One. 2020 Nov 6;15(11):e0242002. doi: 10.1371/journal.pone.0242002 (PMC7647090; doi:10.1371/journal.pone.0242002)
Supplement: S1 Table — (DOC) [file pone.0242002.s001.doc]

| **Nomenclature** | | | | |
| --- | --- | --- | --- | --- |
| **Symbols** | |  | $r$ | variable radius of the Earth |
| $Y_{1}$ | vertical distance from point A to the ground (m) | | $r_{0}$ | average distance of the Earth from the sun throughout the year |
| $X_{1}$ | horizontal distance from point A to the front embankment corner (m) | | $X_{2}$ | horizontal distance from point B to the front embankment corner (m) |
| $a$ | constant | | $P_{1}$ | atmospheric transparency coefficient |
| $b$ | constant | | $I_{W}$ | solar energy captured by the wall (W/m^2^) |
| $Y_{2}$ | vertical distance from point B to the ground (m) | | $I_{\text{g}}$ | solar energy captured by the ground (W/m^2^) |
| $I_{0}$ | solar radiation constant (W/m^2^) | | $J$ | day of the year |
| $I_{T}$ | total solar radiation interceped by the lighting roof (W) | | $t$ | decimal hours on the 24-hour clock |
| $L$ | length of the greenhouse (m) | | $r_{1}$ | light transmission loss caused by the opaque materials |
| $I_{LR}$ | average solar radiation interceped by the lighting roof (W/m^2^) | | $r_{2}$ | light transmission loss caused by the aging of plastic film |
| $I_{LRS}$ | average solar radiation interceped by the lighting roof in sunny day (W/m^2^) | | $r_{3}$ | light transmission loss caused by the dust and water droplets |
| $I_{LRC}$ | average solar radiation interceped by the lighting roof in cloudy day (W/m^2^) | | $T_{O}$ | opening time of the heat preservation quilt (min) |
| $I_{\text{n}}$ | amount of solar direct radiation on the normal plane (W/m^2^) | | $T_{C}$ | closing time of the heat preservation quilt (min) |
| $I_{S}$ | amount of solar diffuse radiation on the normal plane (W/m^2^) | | **Greek symbols** | |
| $h$ | solar altitude angle (°) | | $\tau_{D}$ | direct light transmittance |
| $A_{1}$ | solar azimuth angle (°) | | $\tau_{S}$ | diffuse light transmittance |
| $CCF$ | cloud cover coefficient | | $\tau_{\beta}$ | transmittance at the incident angle of $\beta$ |
| $P$ | constant | | $\tau_{\text{d}}$ | clean pervious to light material of scattered light transmittance |
| $Q$ | constant | | $\sigma$ | solar declination angle (°) |
| $R$ | constant | |  | geographic latitude of the greenhouse (°) |
| $CC$ | amount of the clouds | | $\theta$ | lighting roof angle (°) |
| $r_{n}$ | actual distance of the Earth from the sun on a given day | | $\alpha$ | azimuth angle of greenhouse (°) |
